# Efficacy of electroacupuncture for symptoms of menopausal transition: study protocol for a randomized controlled trial

**DOI:** 10.1186/1745-6215-15-242

**Published:** 2014-06-21

**Authors:** Zhishun Liu, Yang Wang, Huanfang Xu, Jiani Wu, Liyun He, John Yi Jiang, Shiyan Yan, Ruosang Du, Baoyan Liu

**Affiliations:** 1 Guang’anmen Hospital, China Academy of Chinese Medical Sciences, No.5, Beixiange Street, 100053 Beijing, Xicheng District, China; 2 China Academy of Chinese Medical Sciences, No. 16, Nanxiaojie, Dongzhimen Nei, 100700 Beijing, Dongcheng District, China; 3 Acucentre Ltd., 965 Colombo St, Christchurch, New Zealand

**Keywords:** Electroacupuncture, Menopausal transition, Multicenter randomized controlled trial, Safety

## Abstract

**Background:**

Previous studies have shown that acupuncture can alleviate postmenopausal symptoms, such as hot flashes, but few studies have assessed symptoms during the menopausal transition (MT) period. Thus, the effect of acupuncture upon MT symptoms is unclear. We designed a large-scale trial aimed at evaluating the efficacy of electroacupuncture for MT symptoms compared with sham electroacupuncture and at observing the safety of electroacupuncture.

**Methods/design:**

In this multicenter randomized controlled trial, 360 women will be randomized to either an electroacupuncture group or a sham electroacupuncture group. During the 8-week-long treatment, a menopause rating scale, average 24-hour hot flash score, Menopause-Specific Quality of Life Questionnaire score, and level of female hormones will be observed. Follow-ups at the 20th and 32nd week will be made.

**Discussion:**

Though there is no completely inert placebo acupuncture and blinding is difficult in acupuncture trials, the placebo effect of EA can still be partially excluded in this study. For the placebo control, we use non-points and a tailor-made sham needle. This needle is different from a retractable needle, which is usually used for sham acupuncture. The needle in this trial is more simply constructed and more acceptable to Chinese people. We expect to evaluate the efficacy of electroacupuncture for MT symptoms and clarify its effect on these symptoms.

**Trial registration:**

ClinicalTrials.gov Identifier: NCT01849172 (Date of registration: 05/05/2013).

## Background

The menopausal transition (MT) period refers to the time from the commencement of variations in menstrual cycle length and a monotropic rise in follicle-stimulating hormone to the final menstrual period. It is further divided into two phases: the ‘early MT’ in which cycle lengths vary by more than 7 days from the typical length for the individual woman and the ‘late MT’ , characterized by at least two skipped cycles and at least one period of amenorrhea exceeding 60 days [1-4]. Different symptoms appear during different phases of the MT period. Hot flashes are the most frequent symptom and the prevalence of flashes can be as high as 79% at the completion of menopause [5,6]. Other symptoms include night sweats, sleep disturbances, vaginal dryness, urinary incontinence, weight gain, fatigue, irritability, and anxiety. Studies find that most women experience at least one or more of these symptoms as they transit through the postmenopausal stage of life [7].

Hormone therapy (HT) is the recommended drug for women with symptoms related to postmenopause [8]. Because of its long-term risks, HT is no longer prescribed as preventive therapy, but rather for managing menopausal symptoms [9]. Treatment of the perimenopausal woman represents a greater challenge than the menopausal woman because of unpredictable and unstable ovarian function [10]. A study of women’s decision making during the MT has shown that participants believe that menopausal symptoms are best managed with a natural product rather than HT [11].

As part of complementary and alternative medicine, acupuncture has been used in many clinical trials in recent years. Existing studies have shown that acupuncture can alleviate postmenopausal-related symptoms, but we were unable to find many trials on the MT period. A review of acupuncture for treating menopausal hot flashes included six randomized controlled trials (RCTs), which compared acupuncture with sham acupuncture, and they concluded that sham-controlled RCTs failed to show any specific effects of acupuncture for control of menopausal hot flashes [12]. Two trials conducted separately in the United States and South Korea on perimenopausal and postmenopausal women showed acupuncture was beneficial for hot flashes, though it was not more effective than sham acupuncture [13,14]. The limitations of the above two trials were that both trials had small samples (n = 103 and n = 175, respectively), they included both perimenopausal and postmenopausal women, treatment sessions were insufficient (one trial had 10 sessions in 5 weeks and the other 12 sessions in 4 weeks), and follow-up periods were short-term (7 weeks and 4 weeks). Another trial conducted in South Korea reached a similar conclusion that acupuncture failed to show significant effects on hot flash scores compared with sham acupuncture, but it showed partial benefits for hot flash severity [15]. Several trials [16-20] conducted in China demonstrated that electroacupuncture (EA) could reduce the Kupperman Index, follicle-stimulating hormone (FSH), and luteinizing hormone (LH), and increase estradiol (E2), but none of these trials used a sham control or had follow-up. In sum, most trials so far have focused on hot flashes in postmenopausal women and no significant differences were found between acupuncture and sham control. The few trials on MT-related symptoms showed that there was not enough evidence to support acupuncture’s efficacy for MT [21].

We have finished a phase I trial (unpublished), which was a single-blinded (patients blinded) RCT study; 16 women with MT-related symptoms were observed. They were randomized into an EA group (n = 8) and a sham EA group (n = 8). After 8 weeks, the reduction in the menopause rating scale (MRS) was 6.5 ± 9.96 (20.54 ± 14.82 at baseline and 14.00 ± 4.47 after treatment) and 2.00 ± 7.39 (21.50 ± 13.03 at baseline and 19.50 ± 7.85 after treatment) for the EA and sham EA groups, respectively. There was a significant difference between the two groups and the results suggested EA could be beneficial for MT symptoms and have a therapeutic effect.

This protocol is designed for the phase II trial. It is a multi-center randomized placebo controlled trial. It will compare EA with sham EA for symptoms during MT. The primary objective is to evaluate the therapeutic effect of EA for MT symptoms, and to partially exclude the placebo effect. The second objective is to evaluate EA’s safety and any positive effect on the sexual hormone levels (FSH, LH, FSH/LH, and E2) in MT.

## Methods/design

**Figure 1:**
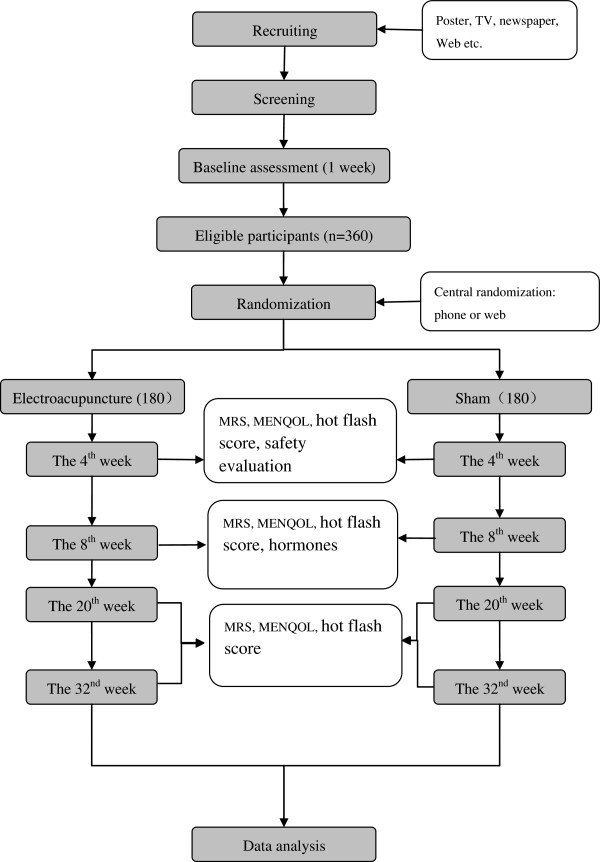
The flow chart.

This will be a prospective RCT conducted over 2 years (from June 2013–May 2015) and at 12 hospitals in China (Guang’anmen Hospital; The First Hospital Affiliated to Anhui University of Chinese Medicine; Guangdong Provincial Hospital of Traditional Chinese Medicine (TCM); Hubei Province Hospital of TCM; The First Hospital of Hunan University of Chinese Medicine; Hengyang Hospital of Hunan University of Chinese Medicine; Affiliated Hospital of Shandong University of TCM; Integrated TCM and Western Medicine of Shanxi University of TCM; Shanxi Province Hospital of TCM; Yueyang Hospital of Shanghai University of TCM; TCM Hospital of Yantai; and 3rd Hospital of Zhejiang Chinese Medical University).Participants experiencing MT will be recruited from the 12 centers by use of advertisements in newspapers, television, websites, and posters. Participants will be sent to a gynecologist who will make the diagnosis. A 1-week baseline assessment will include MRS, Menopause-Specific Quality of Life Questionnaire score (MENQOL), average 24-h hot flash score, and hormone levels (FSH, LH, FSH/LH, and E2, examined at days 2 to 4 of the menstrual period or any day for participants in menopause). Eligible participants will be randomized after their informed consents are obtained. Central randomization will be performed by the Clinical Evaluation Center of the China Academy of Chinese Medical Sciences in Beijing. Stratified block randomization will be made according to the hospital and a block size of six will be used. A random number will be immediately received by the acupuncturist by phone or online after the informed consent is signed. All of the participants, statisticians, outcome assessors, and the gynecologists are blinded to the allocation, except for the acupuncturists. The flowchart of the trial is presented in Figure 1.

### Ethics

The trial will be conducted in accordance with the principles of the Declaration of Helsinki and has been approved by the review boards and ethics committees of the 12 centers (Ethics approval numbers: 2013EC061-01; 2013AH-015; B2013-00084-01; HBZY2013-C025-01; HN-LL-KY-2013-004-01; 2013EC002; 2013020-KY; 2013EC061-01 (20130515); 20130419; 20130620; 2013001; ZSLL-KY-2013-001) (Additional file 1).

### Participants

In total, 360 participants will be recruited in this trial.

#### Inclusion criteria

Participants conforming to all the following conditions will be included: i) cycle irregularity (period occurring 7 days earlier or later) in the past 12 months (early MT) and the last menstruation being at least 2 months previously, but no longer than 11 months previously in the past 12 months (late MT); ii) MT symptoms such as hot flashes, sweating, sleep disturbance, migraine, anxiety, vaginal dryness, and sexual problems; iii) aged 40 to 55 years old; iv) negative pregnancy test; and v) participation is voluntarily and the informed consent signed.

#### Exclusion criteria

Participants with the following conditions will be excluded: i) regular cycles during the past 3 months before enrollment; ii) use of estrogen, serotonin-reuptake inhibitors, soybean isoflavone, progestin, vitamin E, or black sesame in the past 4 weeks; iii) presence of ovarian cyst, uterine myoma (diameter ≥4 cm), or after hysterectomy/ovariectomy; iv) participants with a radiochemotherapy history or undergoing radiochemotherapy; v) cryptogenic vaginal bleeding; vi) coagulation disorder or use of anticoagulants like warfarin and heparin sodium; vii) existing skin diseases like eczema or psoriasis; viii) severe hepatic/renal insufficiency; ix) insufficiently controlled hypertension, diabetes, or thyroid diseases; x) existing diabetic neuropathy, malignant tumor, and psychiatric disorders; xi) desire to become pregnant or pregnant or breast-feeding; xii) regular use of sedatives or anxiolytics; xiii) smoking or heavy alcohol intake; xiv) mandatory indication for HT (e.g., postsurgical menopause or active osteoporosis); and xv) presence of a cardiac pacemaker or artificial joint.

### Interventions

The regimen will be based on a Chinese literature review from the previous 10 years, our phase I trial and specialist’s consensus. Acupuncturists with over 2 years working experience and academic credentials above an undergraduate degree in each hospital will be trained with the protocol before the start of the trial. One session will be given every other day, three sessions per week (24 sessions in all), and each session will last for 30 minutes. A reserved time is made for treatment to prevent participants from communicating with each other.

### Electroacupuncture group

**Figure 2:**
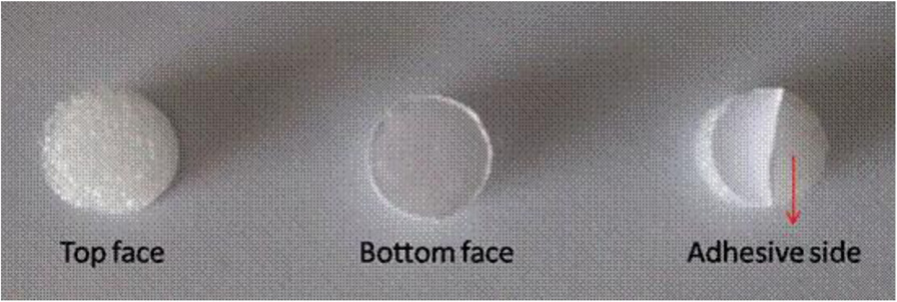
**Adhesive pad.** The adhesive pad used in the trial. The bottom face is adhesive and sticks to the skin.

**Figure 3:**
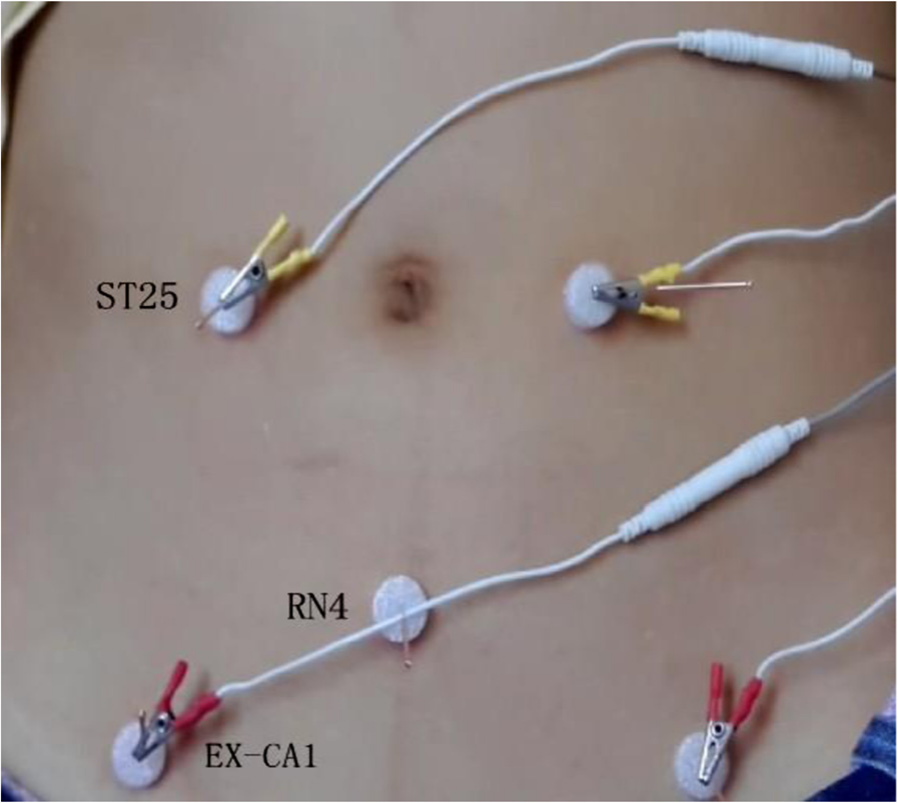
Some points used in the trial.

Acupoints RN4, EX-CA1, ST25, and SP6 on double sides are used. Stick adhesive pads (Figure 2) are placed at all points. For ST25, EX-CA1, and RN4, the needle will be inserted (75 mm) vertically through the pads and quickly through the skin, and then slowly and vertically penetrate through the layer of fatty tissue, up into the muscles of the abdominal wall (until the moment of resistance is sensed on the tip of the needle and the participant feels a sting). For RN4, the needle will be manipulated slightly with an even lifting, thrusting, and twisting method, repeated three times. The needle will be inserted (40 mm) vertically at SP6 to a depth of 1 *cun*. The needle will be manipulated slightly with an even lifting, thrusting, and twisting method repeated three times to reach de-qi. The electric stimulator will be put on the EX-CA1 and ST25 points with a dilatational wave, 10/50 Hz, 0.1 to 1.0 mA. The current intensity will be increased until the abdomen shivers. The needles are left for 30 minutes and the same manipulation methods for RN4 and SP6 will be given every 10 minutes (three times in all) (Figure 3).

### Sham-electroacupuncture group

Non-points proximate to RN4 (P1), EX-CA1 (P2), ST25 (P3), and SP6 (P4) on double sides will be used. P1 and P3 are at the sites 1 *cun* (outward in horizontal direction) proximate to RN4 and ST25, respectively. P2 is 2 *cun* (outward in horizontal direction) proximate to EX-CA1. P4 is at the middle site of the spleen meridian and the kidney meridian (backward in the horizontal direction proximate to SP6). Adhesive pads will be applied to all points. Blunt needles will be inserted but not to pierce the skin.

The needle will be manipulated slightly with an even lifting, thrusting, and twisting method repeated three times for each point. The sham electric stimulator will be put on P2 and P3 with the same parameters as the EA group, but without current intensity. The needles will be left for 30 minutes and the same manipulation methods for all points will be given every 10 minutes. Every point will be checked after treatment to make sure there is no needle hole in the skin.

For participants undergoing bleeding, treatment will be provided. However, for individual participants who do not want needling during their periods, the treatment and outcome assessment will be postponed.

Other MT-relevant treatments will not be allowed throughout the trial. For any use of other treatments, information should be recorded on the case report form in detail. The proportion of participants using other MT-relevant treatment will be compared between groups.

### Outcome

The primary outcome is the change in the MRS compared with baseline at the 8th week (the primary time frame) and at the 4th, 20th, and 32nd week (the secondary time frame). The secondary outcomes include changes in the average 24-h hot flash score [22] (1-week sum score divided by 7, evaluated at the 4th, 8th, 20th, and 32nd week), MENQOL (at the 4th, 8th, 20th, and 32nd week), and FSH, LH, FSH/LH, and E2 (examined at days 2 to 4 of the menstrual period or for participants in menopause, at the end of the 8th week) compared with baseline.

### Safety evaluation and blinding assessment

**Figure 4:**
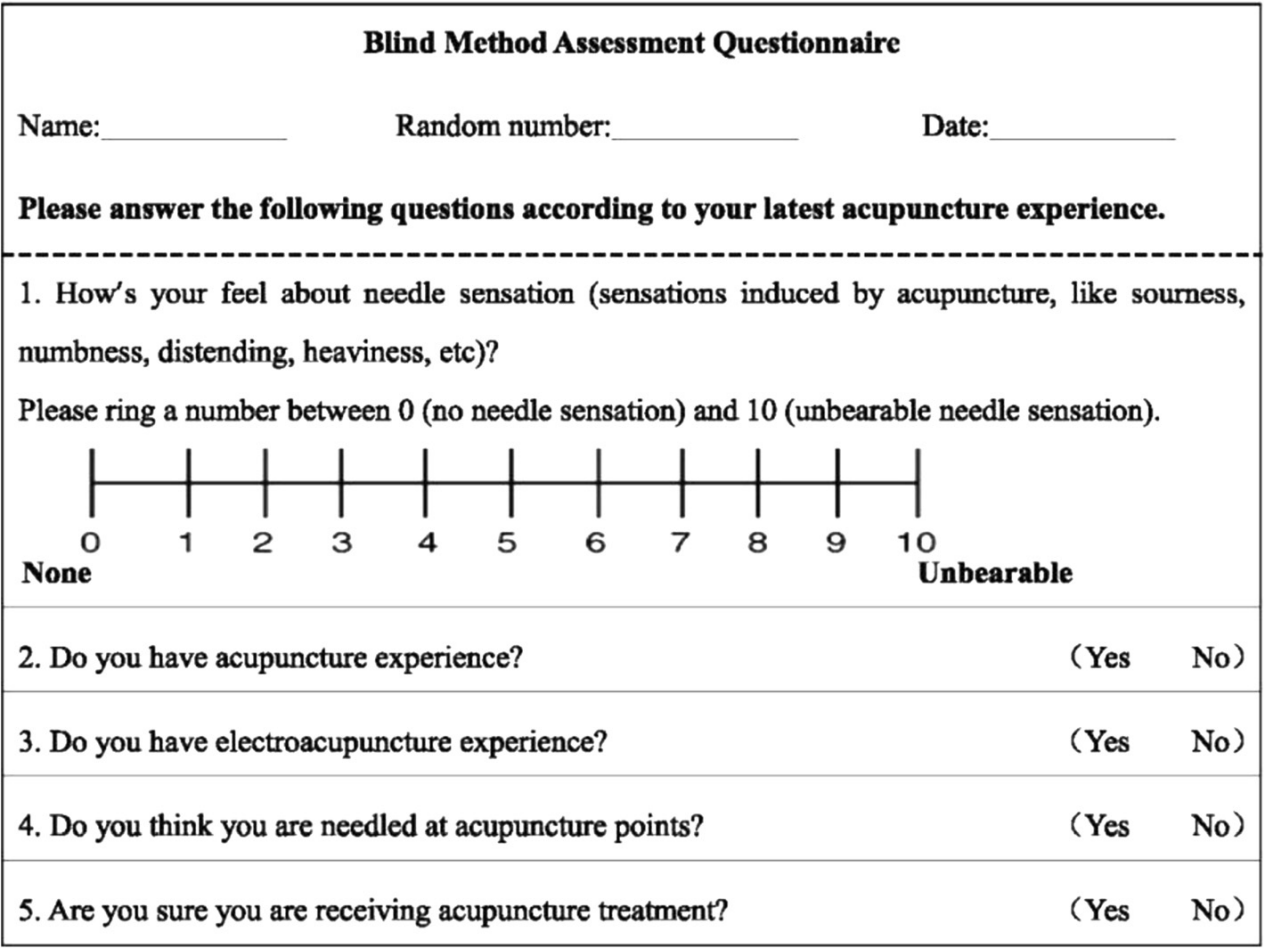
Blinding questionnaire.

Safety evaluation will be based on events including fainting, severe pain, hematoma, local infection, and other feelings of discomfort. Any adverse event will be recorded in detail.To assess whether the sham control is successful, two centers will be randomly chosen to report the EA data by means of a blinded questionnaire (Figure 4). All participants from the two centers will be asked to complete the questionnaire within 5 minutes after any treatment session in the 4th and 8th week. The percentage of participants reporting EA data will be compared between the two groups and success of the blinding will be analyzed.

### Sample size and statistical analysis

According to our pilot trial, the reduction of MRS after an 8-week treatment was 6.5 ± 9.96 (mean ± standard deviation) and 2.00 ± 7.39 for the acupuncture and sham acupuncture groups, respectively. Considering it is a multicenter trial, we expanded the standard deviation to 1.5 times to enhance the reliability of the study. As a minimum 5-point reduction in MRS is considered effective [23], to detect a reduction of 5 points with a two-sided 5% significance level and a power of 95%, 180 participants will be needed for each group (1:1 ratio) allowing for a 20% dropout rate.

Data from the 12 hospitals will be pooled and the Clinical Evaluation Center of China Academy of Chinese Medical Sciences in Beijing will conduct a statistical analysis. According to the principle of intention-to-treat (ITT), all randomized participants will be analyzed. All statistical analyses will be two-sided tests and the level of significance will be established at 0.05. SAS 9.1.3 (SAS Institute, Cary, NC, USA) and SPSS Ver.13.0 (SPSS Inc., Chicago, IL, USA) software will be used. Continuous data will be represented by the mean, standard deviation, median, minimum value, and maximum value; categorical data will be represented by percentages. For comparison with the baseline, a *t*-test or nonparametric test will be used for continuous data, and nonparametric tests for categorical data. For comparison of two independent samples a *t*-test or nonparametric test will be used for continuous data, and a χ^2^ test or the Fisher exact test will be used for categorical data. The multiple imputation method will be used to handle the missing data. The statistical methods are as follows:

1. Case distribution and compliance analysis: case distribution of both groups in every center will be described. The total drop-out rate and the drop-out rate due to adverse events, of both groups, will be compared using the Fisher exact test.

2. Analysis of baseline characteristics.

3. Analysis of compliance: according to recordings in the case report form, compliance of the two groups will be compared.

4. Analysis of efficiency: efficiency analysis will be based on the ITT population (all randomized participants). Subjects without any treatment after randomization or without any valid data of evaluation although treated will still be included in the analysis.

5. Analysis of primary outcome: the primary outcome is the change in MRS compared with baseline at the 8th week (the primary time frame) and at the 4th, 20th, and 32nd week (the secondary time frame). Analysis of covariance (for normal distributed data) or nonparametric tests (for abnormal distributed data) will be used to investigate whether EA is more effective than sham EA. To detect the center effect, a covariance model will be used. Further analysis will be done if there is a center effect.

6. Analysis of secondary outcomes: for each group, the mean 24-h hot flash score, MENQOL, and hormones will be compared with their baseline. A *t*-test or nonparametric test will be used for comparison between groups.

7. Subgroup analysis: subgroup analyses will be conducted according to the stage of MT (early stage and late stage) to observe EA’s influence on MRS and MENQOL at the 4th, 8th, 20th, and 32nd week for participants. Analysis of covariance or nonparametric tests will be used.

8. Analysis of blinding: the number and percentage of subjects choosing true EA in the two selected centers will be established. A χ^2^ test or nonparametric test will be used for comparison among groups.

9. Analysis of safety: according to adverse events listed in the case report form, an analysis will be done using the χ^2^ test or the Fisher exact test.

### Quality control

To guarantee the quality of the trial, a rigorous methodology will be followed. Experts in different fields were invited to review and revise the protocol before the trial start. Staff from the 12 centers will participate in training in Beijing before the trial start. A three-level monitoring system (monitors responsible for one center, monitors responsible for all centers, and monitors responsible for the whole trial) will be established to check the timely performance of the trial. Outcome assessment, completion of case report forms, and data management will be under strict supervision.

## Discussion

In such a large scale RCT, we want to observe EA’s influence on symptoms and quality of life for MT patients. In this trial, not only hot flashes, but also MRS, MENQOL score, and hormone levels will be evaluated. With specific inclusion criteria, only women experiencing MT are included. For the placebo control, we use non-points and a tailor-made sham needle. This needle is different from the retractable needle usually used for sham acupuncture [24,25]. The needle in this trial is more simply constructed and more acceptable to Chinese people. The patent application for the needle is in process. With the use of adhesive pads and a sham electric stimulator, the appearance of the needles and the electric stimulator look exactly alike in the two groups, thus allowing better blinding of the participants. Participants will be treated alone in a treatment room at one time to avoid any communication between them. Though there is no completely inert placebo acupuncture and blinding is difficult in acupuncture trials, the placebo effect of EA can still be partially excluded in this study.

## Conclusions

In summary, the effect of EA for MT symptoms and safety of EA will be assessed in this trial. Following a rigorous set of controls and blinding, the placebo effect should be partially excluded and the trial design should allow for evaluation of the efficacy of EA for MT symptoms.

## Trial status

The first participant was included on 9 June 2013. To date, 80 participants have been recruited.

## Abbreviations

EA: Electroacupuncture; E2: Estradiol; FSH: Follicle-stimulating hormone; HT: Hormone therapy; ITT: Intention-to-treat; LH: Luteinizing hormone; MENQOL: Menopause-specific quality of life questionnaire score; MRS: Menopause rating scale; MT: Menopausal transition.

## Competing interests

The authors declare that they have no competing interests.

## Authors’ contributions

ZL contributed to the conception, design, manuscript writing, and final approval of the manuscript. BL contributed to conception, design, and final approval of the manuscript. LH contributed to conception and final approval of the manuscript. HX contributed to data collection, analysis, and final approval of the manuscript. SY contributed to data analysis and final approval of the manuscript. YW contributed to manuscript writing and final approval of the manuscript. JW contributed to data collection and final approval of the manuscript. RD contributed to data collection, analysis, and final approval of the manuscript. JYJ contributed to manuscript writing, revising data, and final approval of the manuscript. All authors read and approved the final manuscript.

## Supplementary Material

Additional file 1 The name and statement of the 12 research centers’ review boards are listed in the additional file. Click here for file

## References

[B1] Practice Committee of the American Society for Reproductive Medicine The menopausal transition Fertil Steril 2008 90 S61 S65 19007648 10.1016/j.fertnstert.2008.08.095

[B2] Zhang YW Stages of Menopausal Transition. The Second National Menopause Congress 2006 9 12

[B3] Soules MR Sherman S Parrott E Rebar R Santoro N Utian W Woods N Executive summary: Stages of Reproductive Aging Workshop (STRAW) Fertil Steril 2001 76 874 878 10.1016/S0015-0282(01)02909-0 11704104

[B4] Burger H Woods NF Dennerstein L Alexander JL Kotz K Richardson G Nomenclature and endocrinology of menopause and perimenopause Neurotherapeutics 2007 7 Suppl 11 S35 S43 10.1586/14737175.7.11s.S35 18039067

[B5] Woods NF Mitchell ES Symptoms during the perimenopause: prevalence, severity, trajectory, and significance in women's lives Am J Med 2005 118 Suppl 12B 14 24 16414323 10.1016/j.amjmed.2005.09.031

[B6] Williams RE Kalilani L DiBenedetti DB Zhou X Granger AL Fehnel SE Levine KB Jordan J Clark RV Frequency and severity of vasomotor symptoms among peri- and postmenopausal women in the United States Climacteric 2008 11 1 32 43 10.1080/13697130701744696 18202963

[B7] Greenblum CA Rowe MA Neff DF Greenblum JS Midlife women: symptoms associated with menopausal transition and early postmenopause and quality of life Menopause 2012 20 22 27 22929034 10.1097/gme.0b013e31825a2a91

[B8] Sturdee DW Pines A Archer DF Baber RJ Barlow D Birkhauser MH Brincat M Cardozo L de Villiers TJ Gambacciani M Gompel AA Henderson VW Kluft C Lobo RA MacLennan AH Marsden J Nappi RE Panay N Pickar JH Robinson D Simon J Sitruk-Ware RL Stevenson JC Updated IMS recommendations on postmenopausal hormone therapy and preventive strategies for midlife health Climacteric 2011 14 3 302 320 10.3109/13697137.2011.570590 21563996

[B9] The North American Menopause Society Position statement Menopause 2008 15 584 603 10.1097/gme.0b013e31817b076a 18580541 PMC2756246

[B10] Taylor M Alternative medicine and the perimenopause an evidence-based review Obstet Gynecol Clin North Am 2002 29 3 555 573 10.1016/S0889-8545(02)00016-5 12353674

[B11] Theroux R Women’s decision making during the menopausal transition J Am Acad Nurse Pract 2010 22 612 621 10.1111/j.1745-7599.2010.00553.x 21054635

[B12] Lee B-C Shin E Ernst: Acupuncture for treating menopausal hot flushes: a systematic review Climacteric 2009 12 16 25 10.1080/13697130802566980 19116803

[B13] Vincent A Barton DL Mandrekar JN Cha SS Zais T Wahner-Roedler DL Keppler MA Kreitzer MJ Loprinzi C Acupuncture for hot flashes: a randomized, sham-controlled clinical study Menopause 2007 14 1 45 52 10.1097/01.gme.0000227854.27603.7d 17019380

[B14] Kim KH Kang KW Kim DI Kim HJ Yoon HM Lee JM Jeong JC Lee MS Jung HJ Choi SM Effects of acupuncture on hot flashes in perimenopausal and postmenopausal women–a multicenter randomized clinical trial Menopause 2010 17 2 269 280 10.1097/gme.0b013e3181bfac3b 19907348

[B15] Kim DII Jeong JC Kim KH Rho JJ Choi MS Toon SH Choi S-M Kang KW Ahn HY Lee MS Acupuncture for hot flushes in perimenopausal and postmenopausal women: a randomized, sham-controlled trial Acupunct Med 2011 29 249 256 10.1136/aim.2011.004085 21653660

[B16] Xia XH Hu L Qin ZY Zhou J Meng L Li WL Tian LY Zhang YJ Multicentral randomized controlled clinical trials about treatment of perimenopausal syndrome with electroacupuncture of sanyinjiao (SP 6) Acupunct Res 2008 33 4 262 266 18928120

[B17] Jin H Liu TT Wang R Clinical observation on acupuncture at the five-zangshu for treatment of perimenopausal syndrome Chinese Acupuncture Moxibustion 2007 27 8 572 574 17853753

[B18] Qin ZY Ling H Xia XH Meng L Wu ZJ Effects of electroacupuncture of Sanyinjiao (SP 6) on genito-endocrine in patients with perimenopausal syndrome Acupunct Res 2007 32 4 255 259 17907389

[B19] Yan-li HONG Gui-xiang HE Fei WU Cai-ping FANG A randomized controlled study on insomnia during menopause treated with the combination of acupuncture and Chinese herb Luaoning J Tradit Chin Med 2012 39 519 520

[B20] Yanli HONG Guixiang HE Fei WU Clinical study on acupuncture combined with medication for 50 cases of perimenopausal insomnia J Tradit Chin Med 2012 53 1028 1031

[B21] Thacker HL Assessing risks and benefits of non-hormonal treatments for vasomotor symptoms in perimenopausal and postmenopausal women J Womens Health (Larchmt) 2011 20 7 1007 1016 10.1089/jwh.2010.2403 21675874

[B22] Guttuso T Jr DiGrazio WJ Reddy SY Review of hot flash diaries Maturitas 2012 71 3 213 216 10.1016/j.maturitas.2011.12.003 22230663 PMC3275687

[B23] Heinemann LA DoMinh T Strelow F Gerbsch S Schnitker J Schneider HP The Menopause Rating Scale (MRS) as outcome measure for hormone treatment? A validation study Health Qual Life Outcomes 2004 2 67 10.1186/1477-7525-2-67 15555079 PMC534786

[B24] Streitberger K Kleinhenz J Introducing a placebo needle into acupuncture research Lancet 1998 352 364 365 10.1016/S0140-6736(97)10471-8 9717924

[B25] Tan CW Christie L Véronique St-Georges V Telford N Discrimination of real and sham acupuncture needles using the park sham device: a preliminary study Arch Phys Med Rehabil 2009 90 2141 2145 10.1016/j.apmr.2009.08.142 19969182

