# Supplementary material for: Efficacy of electroacupuncture for symptoms of menopausal transition: study protocol for a randomized controlled trial
Source: Trials. 2014 Jun 21;15:242. doi: 10.1186/1745-6215-15-242 (PMC4075980; doi:10.1186/1745-6215-15-242)
Supplement: Additional file 1 — The name and statement of the 12 research centers’ review boards are listed in the additional file. [file 1745-6215-15-242-S1.doc]

**Name and statement of the twelve centers’ review boards**

| No. | Name of hospital | Name of review board | Statement |
| --- | --- | --- | --- |
| 1 | Guang’anmen Hospital | the review boards and ethics committee of Guang’anmen Hospital Affiliated to China Academy of Chinese Medical Sciences | According to Measures for the Ethical Review of Biomedical Research Involving Human Subjects, the Good Clinical Practice, the Guidelines for Ethical Review Work of Drug Clinical Trials, the Guidelines for Ethical Review of TCM Clinical Trials in China, the International Ethical Guidelines  for Biomedical Research Involving Human Subjects and the Declaration of Helsinki, the protocol, informed consent and materials used for recruiting participants are approved. |
| 2 | The First Hospital Affiliated to Anhui University of Chinese Medicine | the ethics committee of The First Hospital Affiliated to Anhui University of Chinese Medicine | According to the Good Clinical Practice (2003), the Guidelines for Ethical Review Work of Drug Clinical Trials (2010), the International Ethical Guidelines for Biomedical Research   Involving Human Subjects (2007), Measures for the Ethical Review of Biomedical Research Involving Human Subjects (2007), Guidelines for Ethical Review of TCM Clinical Trials in China and the Declaration of Helsinki, the protocol and the informed consent are approved. |
| 3 | Guangdong Provincial Hospital of TCM | Institutional Ethics Committee of Guangdong Provincial Hospital of TCM | According to the Good Clinical Practice, Measures for the Ethical Review of Biomedical Research Involving Human Subjects in China, the International Ethical Guidelines for Biomedical Research Involving Human Subjects and the Declaration of Helsinki, the protocol is approved. |
| 4 | Hubei Province Hospital of TCM | Ethics Committee of Hubei Province Hospital of TCM | According to the Good Clinical Practice, Measures for the Ethical Review of Biomedical Research Involving Human Subjects in China, the International Ethical Guidelines for Biomedical Research Involving Human Subjects and the Declaration of Helsinki, the protocol and the informed consent are approved. |
| 5 | The First Hospital of Hunan University of Chinese Medicine | Ethics Committee of the First Hospital of Hunan University of Chinese Medicine | According to the Good Clinical Practice, Measures for the Ethical Review of Biomedical Research Involving Human Subjects in China, the International Ethical Guidelines for Biomedical Research Involving Human Subjects and the Declaration of Helsinki, the protocol, informed consent and materials used for recruiting participants are approved. |
| 6 | Hengyang Hospital of Hunan University of Chinese Medicine | Ethics Committee of Hengyang Hospital of Hunan University of Chinese Medicine | According to Measures for the Ethical Review of Biomedical Research Involving Human Subjects, the Good Clinical Practice, the Guidelines for Ethical Review Work of Drug Clinical Trials, the Guidelines for Ethical Review of TCM Clinical Trials in China, the International Ethical Guidelines  for Biomedical Research Involving Human Subjects and the Declaration of Helsinki, the protocol, informed consent and materials used for recruiting participants are approved. |
| 7 | Affiliated Hospital of Shandong University of TCM | Affiliated Hospital of Shandong University of TCM Ethics Committee | According to the Good Clinical Practice, Measures for the Ethical Review of Biomedical Research Involving Human Subjects in China, the International Ethical Guidelines for Biomedical Research Involving Human Subjects and the Declaration of Helsinki, the protocol and the informed consent are approved. |
| 8 | Integrated TCM and Western Medicine of Shanxi University of TCM | Ethics Committee of Integrated TCM and Western Medicine of Shanxi University of TCM | According to Measures for the Ethical Review of Biomedical Research Involving Human Subjects, the Good Clinical Practice, the Guidelines for Ethical Review Work of Drug Clinical Trials, the Guidelines for Ethical Review of TCM Clinical Trials in China, the International Ethical Guidelines  for Biomedical Research Involving Human Subjects and the Declaration of Helsinki, the protocol, informed consent and materials used for recruiting participants are approved. |
| 9 | Shanxi Province Hospital of TCM | Ethics Committee of Shanxi Province Hospital of TCM | This protocol conforms with the Good Clinical Practice and the Declaration of Helsinki. The trial is allowed to be conducted. |
| 10 | Yueyang Hospital of Shanghai University of TCM | IRB of Yueyang Hospital of Shanghai University of TCM | The trial of electroacupuncture for symptoms of menopausal transition is allowed to be conducted by the review board. |
| 11 | TCM Hospital of Yantai | Ethics Committee of TCM Hospital of Yantai | According to Measures for the Ethical Review of Biomedical Research Involving Human Subjects, the Good Clinical Practice, the Guidelines for Ethical Review Work of Drug Clinical Trials, the Guidelines for Ethical Review of TCM Clinical Trials in China, the International Ethical Guidelines  for Biomedical Research Involving Human Subjects and the Declaration of Helsinki, the protocol, informed consent and materials used for recruiting participants are approved. |
| 12 | 3^rd^ Hospital of Zhejiang Chinese Medical University | Ethics Committee of the 3^rd^ Hospital of Zhejiang Chinese Medical University | The design of the trial conforms with ethical principle and is allowed to be conducted in the hospital. |
